# 15 years journey of idiopathic pulmonary arterial hypertension with BMPR2 mutation

**DOI:** 10.1186/s40885-019-0127-7

**Published:** 2019-10-01

**Authors:** Kyung Jin Ahn, Albert Youngwoo Jang, Su Jung Park, Wook-Jin Chung

**Affiliations:** 1 Gachon Cardiovascular Research Institute, Gachon University, Incheon, Republic of Korea; 2 Department of Pediatrics, Gachon University Gil Medical Center, Incheon, Republic of Korea; 3 Department of Cardiovascular Medicine, Gachon University Gil Medical Center, 21 Namdong-daero 774beon-gil, Namdong-gu, Incheon, 21565 Republic of Korea

**Keywords:** Pulmonary arterial hypertension, Combination therapy, Bone morphogenetic protein receptors, type II

## Abstract

Pulmonary arterial hypertension (PAH) is known as one of diseases with the worst prognosis. Recently, targeted PAH drugs have been developed and approved for use; therefore, the treatment strategy and goals have changed, and the prognosis has improved over two decades. We reviewed the case of a female who showed the natural disease course of heritable PAH in treatment with the targeted PAH drugs under the Korean Health Insurance policy. At the age of 15, she visited the outpatient clinic for dyspnea on exertion that occurred 3 years ago. At that time, severe pulmonary hypertension was revealed by an echocardiography and there was no evidence of significant shunt lesion or embolism. After 4 years of loss to follow-up, her performance was WHO functional class III and she still suffered from dyspnea. The initial monotherapy using an endothelin receptor antagonist was started in 2008. After 2 years, BMPR 2 mutation was detected. Her clinical symptoms gradually worsened because of poor compliance. To escalate therapy, combination therapy was given, and finally, triple maximal therapy was maintained. The next step is to consider intravenous prostanoids. Various combinations of targeted therapy have been tried, and several trials have been confirmed that improve the prognosis. Initial upfront combination therapy and a more enthusiastic approach make good a better prognosis. In this area, active support of the government insurance policy is indispensable in Korea.

## Letter to the Editor

**Fig. 1:**
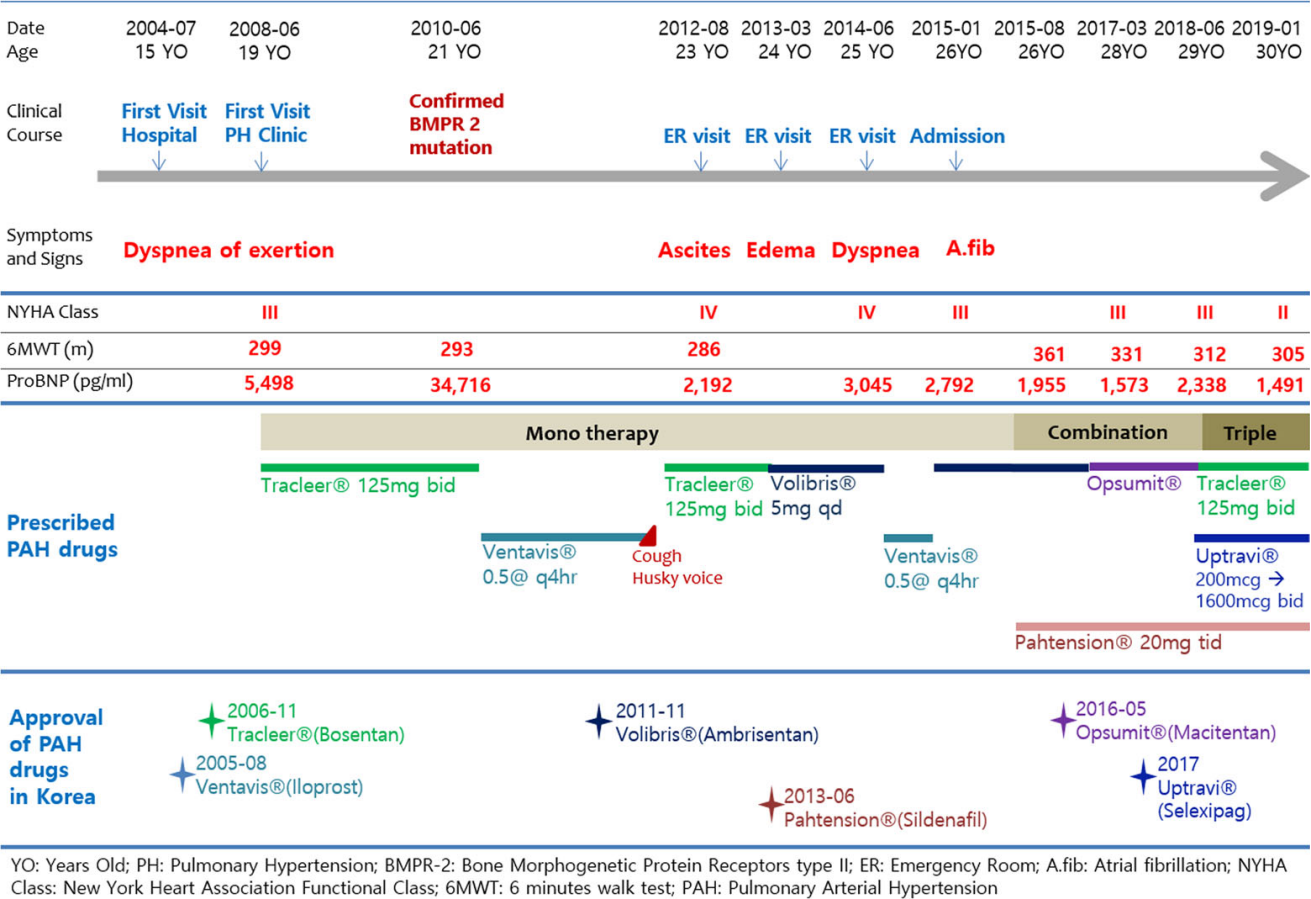
Chronicle of the patient’s clinical features and treatments of PAH specific drugs

**Fig. 2:**
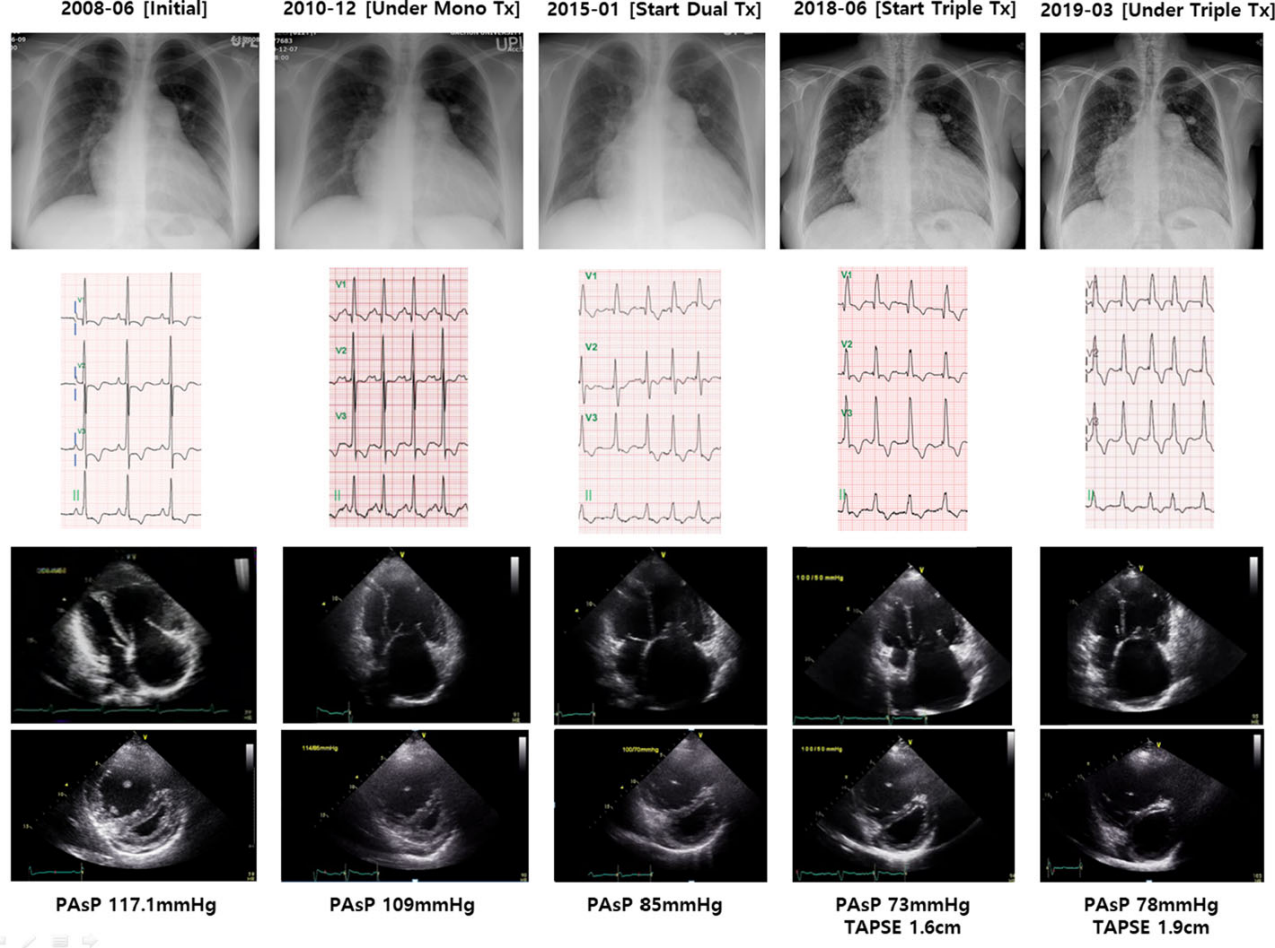
Serial changes of chest radiogram, electrocardiography and trans-thoracic echocardiography

Pulmonary arterial hypertension (PAH) is known to be one of diseases with the worst prognosis. Forty years ago, the American national study reported that the estimated median survival of 194 patients who were diagnosed the primary pulmonary hypertension was 2.8 years [1]. However, the advent of targeted PAH drugs have opened a new era [2, 3]. According to evidence based studies [4–9], the drugs have been developed and approved for use, therefore, the treatment strategy and goals have changed to sequential combination therapy and upfront therapy [10–13]. The prognosis has remarkably improved over two decades [14]. The confidential treatment guidelines are proposed according to the individual risk stratification and the precise diagnosis and classification as deep-phenotyping [15]. However, the guidelines may not be similarly implemented in each country because of each government’s insurance policy. We report here on a case of a female who showed the natural disease course of heritable PAH in treatment with the targeted PAH drugs under the Korean Health Insurance policy. At the age of 15, she visited the outpatient clinic for dyspnea on exertion that initially occurred 3 years before. At that time, severe pulmonary hypertension was revealed by echocardiography and there was no evidence of significant shunt lesion or embolism. After 4 years of loss to follow-up, her performance was WHO Functional Class III as a more aggravated functional state, and she still suffered from severe PAH related symptoms. The right heart catheterization and work-up for risk stratification were performed. The mean pulmonary arterial pressure detected 83 mmHg and the vaso-reactivity tests under inhaled oxygen and iloprost were all negative, respectively. The calculated pulmonary vascular resistance was 2261.6 dyne∙sec∙cm^− 5^. The initial monotherapy, using an endothelin receptor antagonist, was started in 2008. After 2 years, the bone morphogenetic protein receptors (BMPR) type II mutation was confirmed. She carried exon 6 c.631C > T nonsense mutation. Her clinical symptoms gradually worsened because of poor compliance. There were some minor complications as dry cough and changing her voice, but we maintained medications and encouraged the patient. Even though much effort was given, her clinical symptoms deteriorated. To escalate therapy, combination therapy was given and finally triple maximally therapy was maintained (Fig. 1). The serial chest radiogram, electrocardiogram, and trans-thoracic echocardiography showed improvement after sequential combination therapy was administered (Fig. 2). The next step is to consider intravenous prostanoids. Unfortunately, in Korea, we have no further options. To improve prognosis, diagnosis of early disease detection and aggressive early treatment is needed. Especially, intravenous prostanoids are recommended to high risk patients, and are shown to improve outcomes [16]. For example, Japan has a remarkably good prognosis for PAH [17, 18]. What made this outcome possible is the liberal, applicable, targeted drug usage for variable situations to manage PAH patients. Upfront combination therapy and more enthusiastic approaches improve prognosis [19]. In this area, active support of the government insurance policy is indispensable and the most potent factor for improving prognosis. In conclusion, we can propose a good prognosis by appropriate targeted drugs treating the patients with pulmonary hypertension under the supportive government policy.

## Data Availability

Not applicable

## Abbreviations

BMPR 2: Bone morphogenetic protein receptor type 2 gene
PAH: Pulmonary arterial hypertension
WHO: World Health Organization functional class
